# Historical gains in soybean (*Glycine max* Merr.) seed yield are driven by linear increases in light interception, energy conversion, and partitioning efficiencies

**DOI:** 10.1093/jxb/eru187

**Published:** 2014-04-30

**Authors:** Robert P. Koester, Jeffrey A. Skoneczka, Troy R. Cary, Brian W. Diers, Elizabeth A. Ainsworth

**Affiliations:** ^1^Department of Plant Biology, University of Illinois, Urbana-Champaign, 1201W. Gregory Drive, Urbana, IL 61801, USA; ^2^Global Change and Photosynthesis Research Unit, USDA/ARS, Urbana IL 61801, USA; ^3^Department of Crop Sciences, University of Illinois, Urbana-Champaign, 1201W Gregory Drive, Urbana, IL 61801, USA

**Keywords:** Energy conversion efficiency, harvest index, light interception efficiency, partitioning efficiency, radiation use efficiency, yield potential.

## Abstract

Soybean (*Glycine max* Merr.) is the world’s most widely grown leguminous crop and an important source of protein and oil for food and feed. Soybean yields have increased substantially throughout the past century, with yield gains widely attributed to genetic advances and improved cultivars as well as advances in farming technology and practice. Yet, the physiological mechanisms underlying the historical improvements in soybean yield have not been studied rigorously. In this 2-year experiment, 24 soybean cultivars released between 1923 and 2007 were grown in field trials. Physiological improvements in the efficiencies by which soybean canopies intercepted light (ε_i_), converted light energy into biomass (ε_c_), and partitioned biomass into seed (ε_p_) were examined. Seed yield increased by 26.5kg ha^–1^ year^–1^, and the increase in seed yield was driven by improvements in all three efficiencies. Although the time to canopy closure did not change in historical soybean cultivars, extended growing seasons and decreased lodging in more modern lines drove improvements in ε_i_. Greater biomass production per unit of absorbed light resulted in improvements in ε_c_. Over 84 years of breeding, soybean seed biomass increased at a rate greater than total aboveground biomass, resulting in an increase in ε_p_. A better understanding of the physiological basis for yield gains will help to identify targets for soybean improvement in the future.

## Summary

Linear increases in light interception, energy conversion, and partitioning efficiencies have driven past yield gains in soybean. In modern cultivars, canopy light interception and harvest index are reaching theoretical maximum values.

## Introduction

Soybean (*Glycine max*) yields have steadily increased throughout the past century from advances made in breeding, improved management practices, and increased atmospheric carbon dioxide concentrations (Specht *et al.*, 1999; De Bruin and Pederson, 2008; Rowntree *et al.*, 2013). However, the current rate of gain is insufficient to meet the United Nations target of doubling crop yields by 2050 in order to meet the needs of a growing population (Tilman *et al.*, 2011; Ray *et al.*, 2013). While soybean yields have been increased through traditional breeding efforts, the physiological mechanisms underlying past yield gains in the USA are largely unknown. An understanding of the physiological basis of past improvements in soybean yield could help identify strategies for increasing future production.

Yield potential (*Y*_p_) is defined as the maximum yield achieved when a crop is grown in absence of biotic and abiotic stresses (Evans and Fischer, 1999). *Y*_p_ can be parameterized by different efficiencies in the following equation adapted from Monteith (1977):

$$Y_{\text{p}}=\;0.\text{487}S_{\text{t}}\times \;\varepsilon_{\text{i}}\times \;\varepsilon_{\text{c }\times }\varepsilon_{\text{p}}$$

In this equation, *S*_t_ is total incident solar radiation during the growing season of which ~48.7% is photosynthetically active. Light interception efficiency (ε_i_) is determined by the speed and duration of canopy closure along with canopy size and architecture. Energy conversion efficiency (ε_c_), or radiation use efficiency, is determined by the amount of solar energy that is transformed into biomass through the balance of photosynthesis and respiration. Partitioning efficiency (ε_p_), or harvest index, is determined by the amount of biomass energy allocated to vegetative versus reproductive structures (Zhu *et al.*, 2010). The Monteith equation tracks energy transfer from the sun to the seed and provides insight into the physiological mechanisms that ultimately govern yield potential. As a result, the Monteith equation has been used to assess which parameters are at their theoretical maxima and which could be improved further to advance yield (Gifford *et al.*, 1984; Loomis and Amthor, 1999; Reynolds *et al.*, 2000; Reynolds *et al.*, 2010; Zhu *et al.*, 2010; Ainsworth *et al.*, 2012).

The extent to which soybean breeding strategies have improved ε_i_, ε_c_, and ε_p_ in US soybean germplasm has not been investigated. In Chinese and Canadian soybean germplasm, negative correlations between plant height and lodging score with cultivar year of release (YOR) have been reported (Jin *et al.*, 2010; Morrison *et al.*, 2000). These changes in height and lodging improved the standing power of the crop and are hypothesized to increase ε_i_ (Zhu *et al.*, 2010). Improved ε_p_ with YOR in Chinese and Canadian germplasm was attributed to increased seed biomass with little or no increase in total aboveground biomass (Jin *et al.*, 2010; Morrison *et al.*, 1999). There is some evidence that ε_c_ also has been improved by breeding because leaf-level photosynthetic carbon assimilation increased with YOR (Jin *et al.*, 2010; Morrison *et al.*, 1999). However, ε_c_ is the season-long balance between C gain and C loss, and changes in carbon utilization and respiration can offset changes in photosynthesis. Additionally, a direct correlation between leaf-level photosynthesis and crop yield is not consistently apparent (Kumudini, 2002). Therefore, it is not known how decades of soybean breeding have altered ε_c_.

It has been suggested that modern cultivars in high-yielding environments achieve theoretical maximum efficiencies of ε_i_ (0.9) and ε_p_ (0.6), while ε_c_ is far below the theoretical C_3_ maximum (0.094; Zhu *et al.*, 2010). However, there has not been a comprehensive study that parameterizes the Monteith equation across US soybean cultivars with a range of release dates in order to assess how decades of breeding have altered the efficiencies in the field. Further, there is insufficient knowledge about whether elite germplasm are reaching their theoretical maximum efficiencies. Therefore, in order to elucidate the physiological mechanisms of yield improvement in historical soybean germplasm, this study parameterizes the Monteith equation in US soybean cultivars released from 1923–2007. It is hypothesized that: (1) breeding has increased canopy duration and decreased lodging, therefore ε_i_ will increase with cultivar YOR; (2) breeding has improved net C balance in soybean, therefore ε_c_ will increase with cultivar YOR; and (3) seed yield has been increased by traditional breeding while vegetative biomass has not been affected, therefore ε_p_ will increase with YOR.

## Materials and methods

### Experimental design

**Fig. 1.**
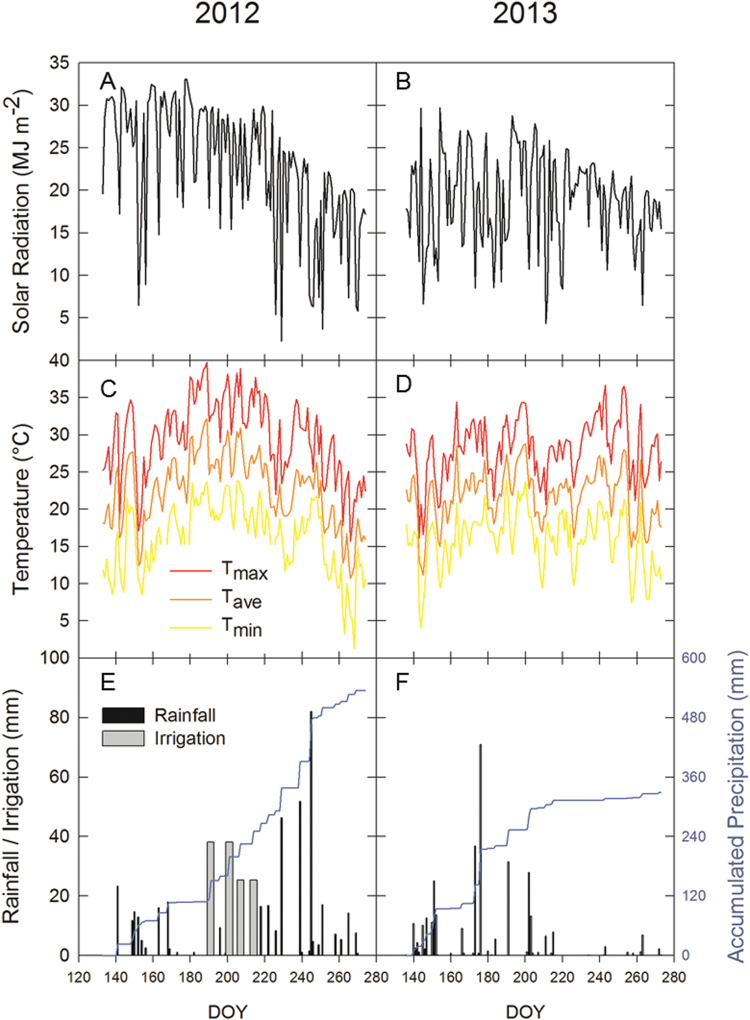
Meteorological data for the 2012 and 2013 experimental growing seasons (planting date to 30 September): daily total solar radiation (A and B), daily maximum, mean, and minimum temperatures (C and D), and rainfall and irrigation events and accumulated precipitation across the growing season (E and F).

Research was conducted at the Crop Research and Education Center in Urbana, IL (40° N 88° 14′W) in 2012 and 2013. Twenty-four indeterminate, maturity group III soybean cultivars were chosen to represent 84 years of past yield gains (Table 1). The publicly developed cultivars were obtained from the USDA Soybean Germplasm Collection, Urbana, IL, courtesy of Dr Randall Nelson. Nonpublic selections were obtained from Pioneer Hi-Bred, Syngenta, and Monsanto and were coded as private entries. Cultivars were chosen to minimize differences in maturity date and to maximize evenness of distribution across the years of study. Seed of all cultivars were produced in a common environment in Illinois the year prior to each experiment. Each year of the experiment was arranged in a randomized complete block design with three replicates. In one block, the cultivars were each grown in large plots (3.05×12.20 m with 16 rows in 2012 and 3.05×9.44 m with 12 rows in 2013) and in the two remaining blocks, cultivars were grown in smaller plots (3.05×3.05 m with four rows in both years). The smaller plots were used to determine seed yield at maturity as well as lodging while the larger plots were used for destructive physiological measurements, tissue sampling, as well as yield determination at maturity. Experimental plots were planted at a row width of 0.76 m and thinned after emergence to a uniform density (Table 2) after unequal stand density was observed in 2011 in a preliminary experiment (Supplementary Fig. S1A available at *JXB* online). Unequal stand density was caused by differences in germination rates (Supplementary Fig. S1B). Daily meteorological data, including *S*_t_ (Fig. 1A, B), temperature (Fig. 1C, D), and precipitation (Fig. 1E, F), were collected ~1.5 km from the field site by the Illinois Climate Network monitoring station (Angel, 2009). Plots were irrigated using drip-line tubing four times during the 2012 season to prevent water stress (Fig. 1E). Drip-line tubing was not laid in 2013 because of ample precipitation early in the growing season.

**Table 1.** List of maturity group III soybean cultivars grown with year of release and plant introduction numberna, not available; PI, plant introduction; YOR, year of release.

Cultivar	YOR	PI no.
Dunfield	1923	PI548318
Illini	1927	PI548348
AK (Harrow)	1928	PI548298
Mandell	1934	PI548381
Lincoln	1943	PI548362
Adams	1948	PI548502
Ford	1958	PI548562
Shelby	1958	PI548574
Ross	1960	PI548612
Adelphia	1964	PI548503
Wayne	1964	PI548628
Calland	1968	PI548527
Williams	1971	PI548631
Woodworth	1974	PI548632
Zane	1984	PI548634
Private 3- 2	1986	na
Resnik	1987	PI534645
Private 3- 9	1989	na
Private 3–19	1994	na
Private 3–11	1996	na
IA 3010	1998	na
IA 3023	2003	na
Private 3–13	2004	na
Private 3–14	2007	na

**Table 2.** Summary of meteorological conditions, plant density, and planting and harvest dates in the 2 years of study

Year	Planting date	Harvest date	Final plant density (plants ha^–1^)	Precipitation (mm)	Mean maximum temperature (°C)	Radiation (MJ m^–2^)
2012	12 May	30 Oct	386,421	483^*a*^	30.6	2944
2013	16 May	14 Oct	379,325	315	28.1	2130

^a^Precipitation plus irrigation.

### Light interception and conversion efficiency

Measurements of ε_i_ were made once or twice per week throughout the growing season. The photosynthetically active radiation (PAR) was measured above (*I*_a_) and below (*I*_b_) the canopy in two undisturbed areas in each large plot between 11:00 and 14:00 on clear-sky days with a 0.87-m line quantum sensor (AccuPAR LP-80, Decagon Devices, Pullman, WA, USA). ε_i_ was estimated from two measurements of PAR directly above the canopy and eight measurements below the canopy. Below-canopy measurements were made ~2.5cm above the ground across a 0.76 m transect between rows. ε_i_ was then calculated as 1 – (*I*_a_/*I*_b_) (Nobel *et al.*, 1993). The season-long mean ε_i_ was calculated using all measurements taken throughout the season. ε_i_ measurements were stopped and assumed to be 0 once the plot reached growth stage R7 defined by pod maturity (Fehr *et al.*, 1971), by which time most of the remaining foliage had senesced.

Aboveground biomass accumulation per unit area was measured every 2 weeks. Avoiding the edges of the plot (0.5 m), a 1-m length of row was harvested at 2.5cm above the ground. Plants were counted and separated into leaf, stem (including petioles and petiolules), and pod sections. Plant material was then dried for 1 week at 70 °C and weighed. In order to convert total biomass into energy equivalents, seeds, leaves, and stems were ground and analysed for total energy content using adiabatic bomb calorimetry (model 1261, Parr Instrument, Moline, IL, USA) with benzoic acid as a standard (Supplementary Figs S2 and S3). Biomass measurements were made in parallel with ε_i_ measurements. Cumulative intercepted radiation (PAR_i_) at the time of each biomass harvest was calculated by multiplying the accumulated PAR by the linearly interpolated ε_i_ estimated for each period of time between biomass harvests. For calculation of season-long ε_c_, cumulative PAR_i_ (MJ m^–2^) was plotted against cumulative biomass energy (MJ m^–2^) until peak biomass was observed. The slope of the linear fit was used to estimate ε_c_ (Monteith, 1972) and it was assumed that ε_i_ was 0 on the day of crop emergence.

### Partitioning efficiency and yield

ε_p_ was calculated as the ratio of seed biomass to total aboveground biomass and also expressed in terms of energy content of the seed to the energy content of total aboveground biomass at full maturity (R8; Fehr *et al.*, 1971). Total seed and stem biomass was measured as afore described, except 2 m of row were harvested for calculation of ε_p_. Lodging scores were determined in all three experimental plots using a 0–10 scale according to the following system: most main stems were completing vertical at 0° (0), 45° (5), completely horizontal at 90° (10). When the cultivars had reached maturity, yield was determined by harvesting two centre rows from each of the three yield plots with a 2-row combine and estimates were adjusted to 13% seed moisture content.

### Statistical analysis

A significant correlation between yield, Monteith efficiencies, and cultivar YOR was tested using least-squares regressions (PROC MIXED procedure, SAS version 9.2, SAS Institute, Cary, NC, USA) or first-order linear regression (SigmaPlot, Systat Software, Richmond, CA, USA). A t-test was used to determine if linear regressions slopes were significantly different among years. A two-segment linear regression model (PROC NLMIXED procedure, SAS version 9.2) was also fit to the data and compared to the linear fit using the Akaike information criterion coefficient.

## Results

### Yield increased linearly with cultivar YOR

**Fig. 2.**
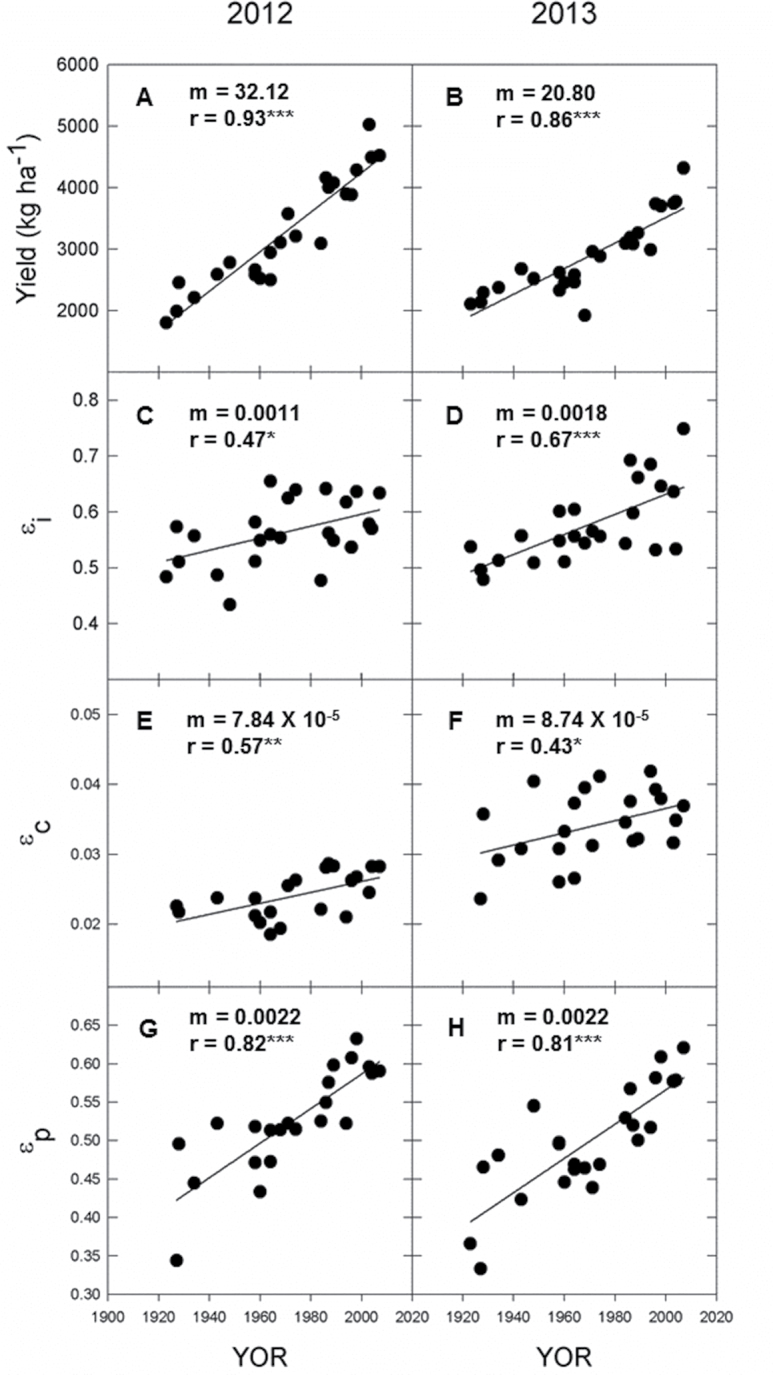
Seed yield, ε_i_, ε_c_, and ε_p_ with soybean cultivar year of release (YOR) for the 2012 and 2013 growing seasons: seed yield (A and B), seasonal interception efficiency (ε_i_, C and D), conversion efficiency (ε_c_, E and F), and partitioning efficiency expressed in energy content (ε_p_, G and H). Lines represent significant least-squares regression. *m*, slope; *r*, Pearson correlation coefficient; *, **, and *** denote significance at *P*<0.05, *P*<0.01, and *P*<0.001, respectively.

There was a linear improvement in soybean yields with cultivar YOR, with increases of 32.1kg ha^–1^ year^–1^ in 2012 and 20.8kg ha^–1^ year^–1^ in 2013 (Fig. 2A, B). The rate of yield gain in 2012 was significantly greater than in 2013 (*P*<0.005). Older cultivars showed less year-to-year variation in seed production, with yield differences of ~145kg ha^–1^ between years, while the newest cultivars yielded ~800kg ha^–1^ more in 2012 compared to 2013 (Fig. 2A, B). Newer cultivars (Private 3–14, Private 3–13. and IA3023) were consistently among the highest yielding and older cultivars (Dunfield and Illini) were the lowest in both years of the experiment.

### ε_i_ increased with cultivar YOR

**Fig. 3.**
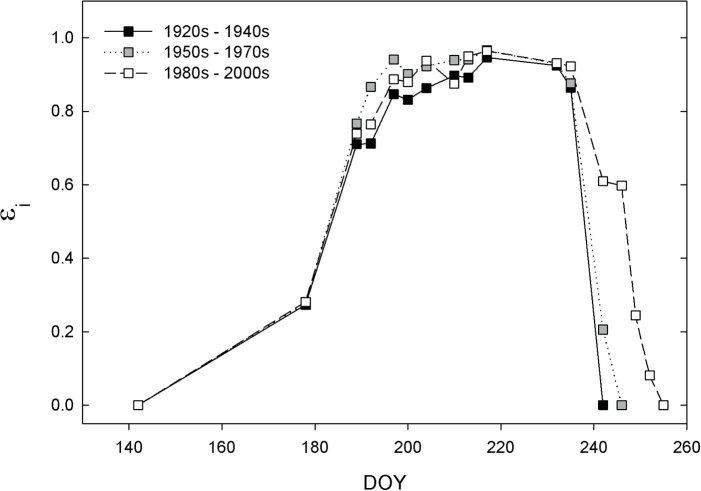
Interception efficiency (ε_i_) across the growing season in 2013 for each of the 24 soybean cultivars grouped by YOR. DOY, day of year.

Season-long ε_i_ increased with YOR in both 2012 and 2013 (Fig. 2C, D), and the slopes in the trends were not significantly different between years (*P*=0.24). Increases in season-long ε_i_ with cultivar YOR were driven by a longer growing season, with more recent cultivars maturing later (Fig. 3). The growing season was ~10 d longer in lines released from the 1980s to the 2000s, compared to the lines released from the 1920s to the 1940s (Fig. 3). There was difference in the rate of canopy closure in older or newer cultivars, and most cultivars approached 90% closure by ~60 d after planting (Fig. 3). Lodging, which negatively affects ε_i_ at the end of the growing season, also decreased with YOR (Supplementary Fig. S4).

### ε_c_ increased with cultivar YOR

ε_c_ increased with cultivar YOR in 2012 and 2013 (Fig. 2E, F). In 2012, cultivars released between 1990 and 2000 accumulated 14.1 MJ m^–2^ in biomass over the growing season, compared to 12.9 MJ m^–2^ in cultivars released between 1920 and 1940. Similarly, in 2013, cultivars released between 1990 and 2000 accumulated 17% more biomass over the growing season compared to cultivars released between 1920 and 1940.

**Fig. 4.**
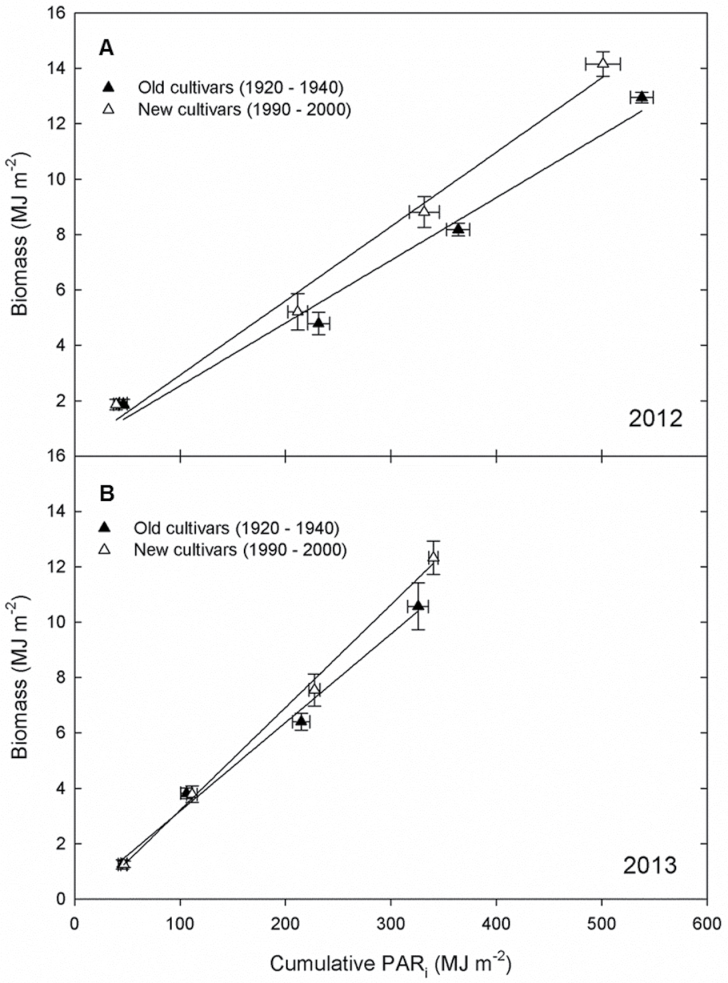
Accumulated aboveground biomass versus cumulative PAR_i_ in 2012 (A) and 2013 (B). Lines represent least-squared regression between dry biomass versus cumulative PAR_i_. The slope of each line (*m*) is ε_c_. Each point represents the biomass and cumulative PAR_i_ for the five oldest cultivars and the five most recently released cultivars.

While the slopes of the trends in ε_c_ with cultivar YOR did not significantly differ between years, ε_c_ was significantly greater in 2013 compared to 2012 (Fig. 2E, F). This was driven primarily by differences in accumulated PAR in the two years. In 2012, cultivars accumulated ~13% more total peak biomass than in 2013, but did so by using 33% more intercepted PAR, resulting in lower values of ε_c_ (Fig. 4).

### ε_p_ increased with cultivar YOR

**Fig. 5.**
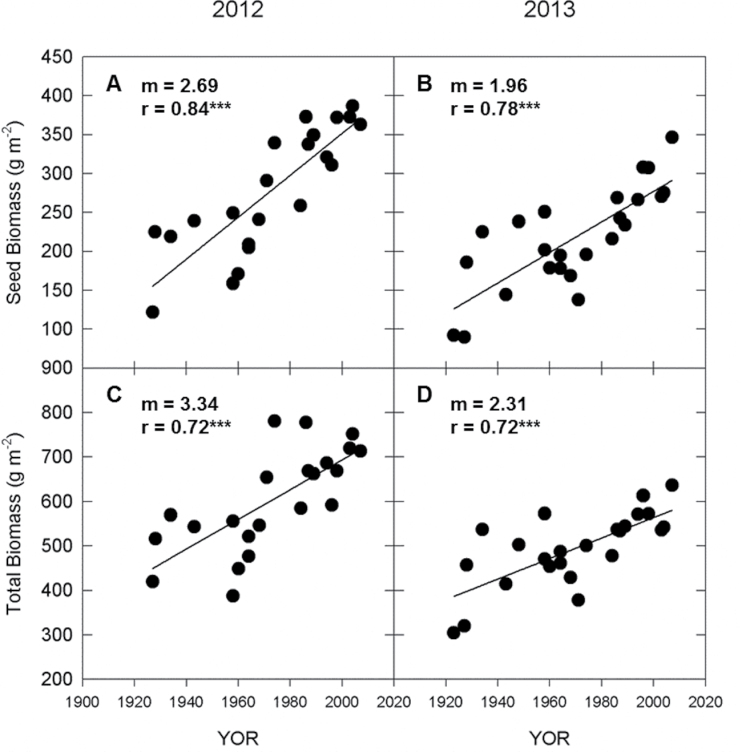
Determinants of partitioning efficiency (ε_p_) versus YOR at growth stage R8 plotted against cultivar YOR in 2012 and 2013: seed biomass (A and B) and total biomass (C and D). Lines represent significant least-squares regression (*** *P*<0.001).

ε_p_ expressed on an energy basis increased significantly with cultivar YOR in both years of the study (Fig. 2G, H). Gains in ε_p_ were driven primarily by increases in total seed biomass as ~80% of the gain in total biomass was caused by increases in seed biomass (Fig. 5). Although the values of seed and total biomass were greater in 2012 compared to 2013 (Fig. 5), the ratio of seed to total biomass was similar and therefore the rate of gain in ε_p_ with YOR was the same in both years (Fig. 2G, H).

### Yield correlations with Monteith efficiencies

**Fig. 6.**
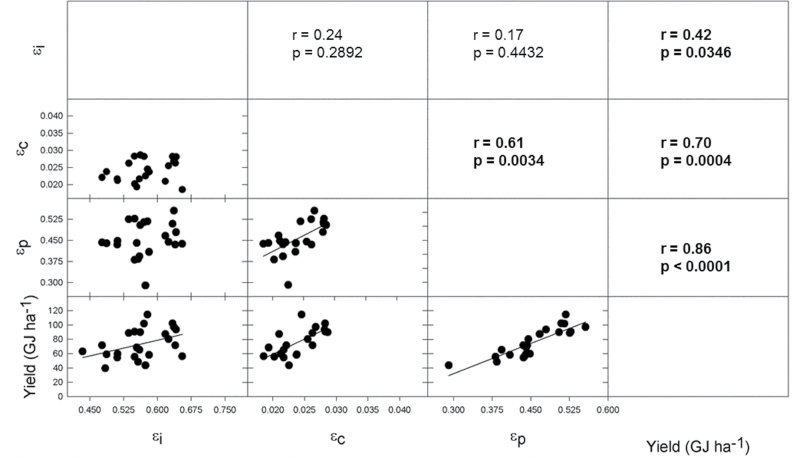
2012 correlation matrix of yield and Monteith efficiencies. ε_p_ is expressed in terms of biomass (g seed/g total aboveground biomass). Scatterplots and correlation coefficients are plotted in a matrix where lines represent significant least-squares regression. Bold indicates significant results.

**Fig. 7.**
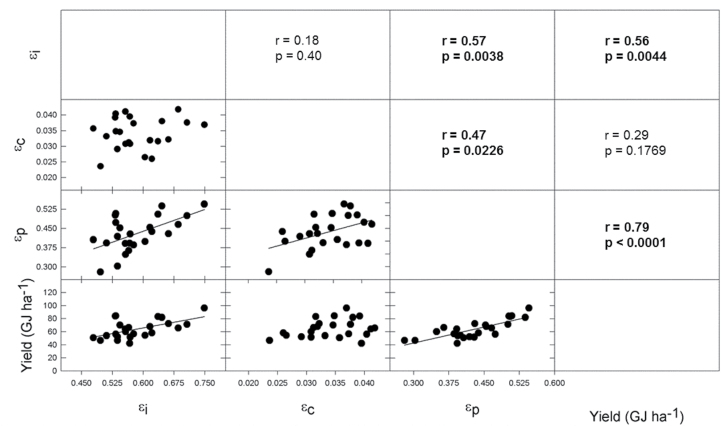
2013 correlation matrix of yield and Monteith efficiencies. ε_p_ is expressed in terms of biomass (g seed/g total aboveground biomass). Scatterplots and correlation coefficients are plotted in a matrix where lines represent significant least-squares regression. Bold indicates significant results.

In 2012, all three Monteith efficiencies (ε_i_, ε_c_, and ε_p_) were significantly correlated with yield (Fig. 6), and ε_c_ and ε_p_ were correlated with one another (Fig. 6). However, ε_i_ was not correlated with ε_c_ or ε_p_ in 2012. In 2013, ε_i_ and ε_p_ were significantly correlated with yield (Fig. 7) but ε_c_ was not (Fig. 7). ε_i_ was more strongly correlated to yield in 2013 (Fig. 7), a year with ~30% less total solar radiation over the growing season compared to 2012. ε_p_ is autocorrelated with seed yield and therefore it showed very high correlation coefficients in both years (Figs 6 and 7).

## Discussion

In field trials of US soybean cultivars released over the past 84 years, seed yield significantly increased with YOR. When separating yield into its physiological efficiencies, there were consistent increases in the efficiencies by which canopies intercepted solar energy, converted it into biomass, and partitioned biomass into yield. In a highly productive agricultural area in the midwest USA, peak ε_i_ is >90% and ε_p_ is reaching the theoretical maxima value (60%) in recently released soybean cultivars. However, there is still room for further improvement in ε_c_ in modern soybean cultivars.

This study of historical soybean cultivars estimated rates of soybean yield gain of 1.8% year^–1^ in 2012 and 1.1% year^–1^ in 2013. These rates are in line with the annual percentage gains reported in a literature review by Specht *et al.* (1999) and are similar to rates reported in a recent study of 60 cultivars with a similar range of YOR dates that also included the 24 cultivars grown in this present study (Rowntree *et al.*, 2013; Rincker *et al.*, 2014). While Rincker *et al.* (2014) found the data were better described by a two-segment linear fit with different slopes before and after 1964, the rates of yield gain in this study were better described by a single linear fit, perhaps because there was less power in this study to detect differences in the rate of yield gain before and after 1964. The gains in soybean yield reported here are also similar to improvements reported for other major crops including maize (*Zea mays*; Duvik and Cassman, 1999; Richards, 2000), rice (*Oryza sativa*; Peng *et al.*, 2000), and wheat (*Triticum aestivum*; Reynolds *et al.*, 1999; Shearman *et al.*, 2005). The greater rates of yield gain observed in 2012 compared to 2013 were likely caused by differences in environmental factors and irrigation. The experimental site experienced hot, dry growing conditions in 2012, so plots were irrigated to reduce water stress. The 2013 growing season had lower maximum temperatures, less incoming solar radiation, and ample water early in the season. However, drought conditions occurred after the canopy closed and when seeds were filling, which likely contributed to the lower rate of gain in seed yield in 2013. When comparing the 2 years of the study, it was also notable that more recently released cultivars showed greater yields in the more favourable growing environment in 2012, while older cultivars had more consistent yields in 2012 and 2013. This result suggests that although newer cultivars consistently outperformed older cultivars in all environments, they may have greater environmental sensitivity. These results are consistent with Rincker *et al.* (2014), who found greater rates of soybean yield gain in high-yielding environments and lower yield stability in more recently released cultivars.

The effective capture of solar radiation across the growing season determines how much solar energy is available for conversion into biomass and therefore yield. In this study, ε_i_ increased with cultivar YOR similarly across both years, with soybean canopies intercepting approximately 50–75% of the growing season’s PAR. Peak ε_i_ in all soybean cultivars was >90%, consistent with previous reports (Dermody *et al.*, 2008). However, the seasonal ε_i_ measured in this study is lower than the theoretical maximum ε_i_ for soybean of ~90% (Zhu *et al.*, 2010) and lower than previously reported levels of 89% (Dermody *et al.*, 2008). This may be because the current study used wider row spacing than Dermody *et al.* (2008) and because the current study took more measurements early in the growing season when the canopy was still developing. There was no difference in time to canopy closure among new and old soybean varieties, but rather an increase in the duration of a photosynthetically active canopy allowing greater capture of *S*_t_. This was in part because more recent cultivars have later maturity dates (Rowntree *et al.*, 2013) but also because lodging significantly decreased with YOR, which lengthened the duration of an active canopy. Other studies in soybean have reported similar improvements in lodging score over years of breeding (Specht *et al.*, 1999; Morrison *et al.*, 2000; Jin *et al.*, 2010). There are very few direct estimates of ε_i_ in soybean, but leaf area index (LAI) is commonly measured and used to indicate ε_i_. A decreasing trend in LAI with YOR has been reported (Morrison *et al.*, 1999; Jin *et al.*, 2010), indicating that newer cultivars with lower LAI may have reduced capacity for intercepting light. However, while LAI can be a good indicator of light interception at the early stages of canopy closure, at an LAI of 3.5–4.0 light interception exceeds 95% (Board and Harville, 1992). Therefore, LAI values above ~4.0 reveal very little about ε_i_. Improvement strategies for light interception in major crops tend to focus primarily on extending the growing season and/or engineering for optimal crop canopy architecture (Reynolds *et al.*, 2000; Parry *et al.*, 2010; Zhu *et al.*, 2010), which would increase the total *S*_t_ for the crop to intercept. In rice, for example, each day added to the growing season translated into a 180kg ha^–1^ increase in yield (Akita, 1988).

Energy conversion efficiency and its improvement has been the focus of many yield improvement strategies (Amthor, 2010; Zhu *et al.*, 2010; Parry *et al.*, 2010; Raines, 2011; Ainsworth *et al.*, 2012). Yet the extent of how ε_c_ has been improved through historical breeding is not well understood. In this study, ε_c_ increased with YOR in both 2012 and 2013, leading to a ~36% improvement over the 84 years covered in this study (~0.43% year^–1^). A similar increase in ε_c_ in wheat cultivars released from the 1970s to the 1990s has been reported (Shearman *et al.* 2005); however, earlier studies of different wheat cultivars failed to report a similar trend (Slafer *et al.*, 1990; Calderini *et al.*, 1997). In the current study, ε_c_ was 29% higher in 2013 compared to 2012, with a maximum ε_c_ of 2.9% in 2012 and 4.3% in 2013. These rates are higher than the rates of field-grown C_3_ crops (2.4%) previously reported by Zhu *et al.*, (2008) but still well below the theoretical maximum of 9.4% (Zhu *et al.*, 2010). The exclusion of root biomass in the calculation of ε_c_ also underestimates the efficiency (Sinclair and Muchow, 1999), although it is not known how traditional breeding has affected root biomass allocation. ε_c_ is estimated from the linear relationship between biomass accumulation and intercepted light, and gains in ε_c_ in recently released soybeans came from increased biomass production for a given amount of intercepted light (Fig. 4). Changes in respiration or photosynthesis could underpin this trend in ε_c_, and previous work in Canadian and Chinese germplasm suggests that leaf-level photosynthesis has improved with YOR (Jin *et al.*, 2010; Morrison *et al.*, 1999). However, future studies are needed to determine the mechanisms driving improvements in ε_c_ in these maturity group III historical lines. ε_c_ in 2012 was lower than in 2013, because although the crop intercepted 33% more PAR in 2012 than in 2013, peak biomass was only 13% greater in 2012 than 2013. Photosynthesis saturates at ~50% full sunlight and plants are not able to utilize all the intercepted solar radiation, which results in decreased efficiencies of energy conversion (Sinclair and Muchow, 1999; Ort, 2001). A recent meta-analysis by Slattery *et al.* (2013) found in shading experiments that ε_c_ increased by 18% when plants were grown in shaded conditions compared to full sunlight. Consistent with the meta-analysis, ε_c_ of soybean was greater in a year with less solar radiation; however, despite the increased efficiency in 2013, 2012 resulted in higher absolute seed yields. Although the plants were less efficient in the amount of C fixed per MJ of light in 2012, the plants had higher rates of incident solar radiation throughout the growing season which more than compensated for the loss of efficiency and led to the increase in peak biomass.

Consistent increases in ε_p_ with YOR were observed in 2012 and 2013. The range of ε_p_ based on biomass for both years was similar (0.3–0.55), and the most recently released cultivars approached the theoretical maximum of 0.60 (Figs 6 and 7). The improvement of ε_p_ with YOR was achieved through tripling seed biomass per area but only doubling total biomass per area (Fig. 5). The rate of gain in ε_p_ in Chinese soybean germplasm was similar at 0.40% year^–1^ (Jin *et al.*, 2010). In Canadian soybean germplasm, historical improvements in ε_p_ were only due to increases in seed weight and not total biomass (Morrison *et al.*, 1999). In other major food crops, particularly small grains, improvements in ε_p_ largely drove improvements in yield from 1900 to 1980 (Hay, 1995). In wheat, linear increases in ε_p_ were found with YOR in the UK and Mexico and were achieved through increased grain yield with no increase in total biomass (Austin *et al.*, 1989; Sayre *et al.*, 1997). More recently, Shearman *et al.* (2005) reported that ε_p_ levelled off at ~0.5 when they looked at cultivars of wheat that were released from 1970 to 1995. Historically, rice showed improvements in ε_p_ until it reached a maximum of around 0.6 in the 1980s when increases in yield were then attributed to greater rates of biomass production (Hay, 1995; Peng *et al.*, 2000). The ε_p_ of maize was already high (~0.45) in the early 1930s and therefore gains in maize yield were made through increases in total biomass (Hay, 1995; Richards, 2000). While the data presented here support a linear increase in ε_p_ with YOR in soybean (i.e. the data are not reaching a plateau), ε_p_ in the most recently released lines is approaching the theoretical maximum.

The contribution to yield gain by the Monteith efficiencies was investigated by analysing their correlations with yield. ε_p_ is autocorrelated with yield and so it not surprisingly showed the strongest correlations in both 2012 and 2013 (Figs 6 and 7). Yield correlations with ε_i_ and ε_c_ were more variable and weaker. In both years of the study, ε_i_ correlated significantly with yield whereas ε_c_ only correlated with yield in 2012. Interestingly, there was no correlation between ε_i_ and ε_c_, suggesting that the improvements in these traits in historical germplasm may have been independent. The correlations with yield suggest that improvements in all Monteith efficiencies were important to past yield gains, and they are all targets of international efforts to improve future C_3_ crop yields (Reynolds *et al.*, 2010).

In conclusion, several physiological changes have accompanied the impressive gains in soybean yield over the past 80 years. First, soybean canopies of more recently released cultivars have greater season-long canopy interception efficiencies owing to longer growing seasons and improved resistance to lodging. Second, modern soybean cultivars have better efficiencies of converting light energy into aboveground biomass and produce 9–17% more aboveground biomass energy in a growing season than cultivars released before 1950. Third, the partitioning of biomass to seeds has been maximized in modern soybean lines. Where is there room for future improvement in soybean yield? Longer growing seasons would enable already efficient soybean canopies to harvest more light (Rowntree *et al.*, 2013), but there appears to be little room for improving ε_p_. On the other hand, ε_c_ is still well below the theoretical maximum, even in the most recently released cultivars, and therefore it is an important target for future improvement.

## Supplementary material

Supplementary data are available at *JXB* online.

Supplementary Fig. S1. Plant density and seed germination versus YOR in 2011.

Supplementary Fig. S2. Leaf and stem energy content versus YOR.

Supplementary Fig. S3. Seed composition versus YOR in 2012 and 2013.

Supplementary Fig. S4. Lodging score versus YOR in 2012 and 2013.

Supplementary Data

## References

[CIT0001] Ainsworth EA Yendrek CR Skoneczka JA Long SP 2012 Accelerating yield potential in soybean: potential targets for biotechnological improvement. Plant, Cell and Environment 35, 38–52 10.1111/j.1365-3040.2011.02378.x 21689112

[CIT0002] Akita A 1988 Physiological basis of heterosis in rice. In: Hybrid rice. Proceedings of the International Symposium on Hybrid Rice. Los Banos, Phillipines: International Rice Research Institute pp 67–77

[CIT0003] Austin RB Ford MA Morgan CL 1989 Genetic improvement in the yield of winter wheat: a further evalutation. Journal of Agricultural Science 112, 295–301

[CIT0004] Amthor JS 2010 From sunlight to phytomass: on the potential efficiency of converting solar radiation to phyto-energy. New Phytologist 188, 939–959 20977480 10.1111/j.1469-8137.2010.03505.x

[CIT0005] Angel J 2009 The Water and Atmospheric Resources Monitoring Program. Urbana, IL: Illinois State Water Survey, University of Illinois at Champaign-Urbana

[CIT0006] Board JE Harville BG 1992 Explanations for greater light interception in narrow- vs. wide-row soybean. Crop Science 32, 198–202

[CIT0007] Calderini DF Dreccer MF Slafer GA 1997 Consequences of plant breeding on biomass growth, radiation interception, and radiation use efficiency in wheat. Field Crops Research 52, 271–281

[CIT0008] De Bruin JL Pederson P 2008 Yield improvement and stability for soybean cultivars with resistance to *Heterodera glycines* Ichinohe. Agronomy Journal 100, 1354–1359

[CIT0009] Dermody O Long SP McConnaughay K DeLucia EH 2008 How do elevated CO_2_ and O_3_ affect the interception and utilization of radiation by a soybean canopy? Global Change Biology 14, 556–564

[CIT0010] Duvik DN Cassman KG 1999 Post-green revolution trends in yield potential of temperate maize in the north-central United States. Crop Science 39, 1622–1630

[CIT0011] Evans LT Fischer RA 1999 Yield potential: its definition, measurement, and significance. Crop Science 39, 1544–1551

[CIT0012] Fehr WR Caviness CE Burmood DT Pennington JS 1971 Stage of development descriptions for soybeans, *Glycine max* (L.) Merr. Crop Science 11, 929–931

[CIT0013] Gifford RM Thorne JH Hitz WD Giaquinta RT 1984 Crop productivity and photoassimilate partitioning. Science 225, 801–808 17801136 10.1126/science.225.4664.801

[CIT0014] Hay RKM 1995 Harvest index: a review of its use in plant breeding and crop physiology. Annals of Applied Biology 126, 197–216

[CIT0015] Jin J Liu K Wang G Mi L Shen Z Chen X Herbert SJ 2010 Agronomic and physiological contributions to the yield improvement of soybean cultivars released from 1950 to 2006 in Northeast China. Field Crops Research 115, 116–123

[CIT0016] Kumudini S 2002 Trials and tribulations: a review of the role of assimilate supply in soybean genetic yield improvement. Field Crops Research 75, 211–22

[CIT0017] Loomis RS Amthor JS 1999 Yield potential, plant assimilatory capacity, and metabolic efficiencies. Crop Science 39, 1584–1596

[CIT0018] Monteith JL 1972 Solar radiation and productivity in tropical ecosystems. Journal of Applied Ecology 9, 747–766

[CIT0019] Monteith JL 1977 Climate and the efficiency of crop production in Britain. Philosophical Transactions of the Royal Society B 281, 277–294

[CIT0020] Morrison MJ Voldeng HD Cober ER 1999 Physiological changes from 58 years of genetic improvement of short-season soybean cultivars in Canada. Agronomy Journal 91, 685–689

[CIT0021] Morrison MJ Voldeng HD Cober ER 2000 Agronomic changes from 58 years of genetic improvement of short-season soybean cultivars in Canada. Agronomy Journal 92, 780–784

[CIT0022] Nobel PS Forseth IN Long SP 1993 Canopy structure and light interception. In: Hall DO Scurlock JMO Bolhar-Nordenkampf HR Leegood RC Long SP, eds, Photosynthesis and production in a changing climate. London: Chapman and Hall pp 79–90

[CIT0023] Ort DR 2001 When there is too much light. Plant Physiology 125, 29–32 11154289 10.1104/pp.125.1.29 PMC1539318

[CIT0024] Parry MA Reynolds M Salvucci ME Raines C Andralojc PJ Zhu X-G Price GD Condon AG Furbank RT 2010 Raising yield potential of wheat. II. Increasing photosynthetic capacity and efficiency. Journal of Experimental Botany 62, 453–467 21030385 10.1093/jxb/erq304

[CIT0025] Peng S Laza RC Visperas RM Sanico AL Cassman KG Khush GS 2000 Grain yield of rice cultivars and lines developed in the Philippines since 1966. Crop Science 40, 307–314

[CIT0026] Raines CA 2011 Increasing photosynthetic carbon assimilation in C_3_ plants to improve crop yield: current and future strategies. Plant Physiology 115, 36–42 21071599 10.1104/pp.110.168559 PMC3075778

[CIT0027] Ray DK Mueller ND West PC Foley JA 2013 Yield trends are insufficient to double global crop production by 2050. PLoS ONE 19, e66428 23840465 10.1371/journal.pone.0066428 PMC3686737

[CIT0028] Reynolds M Bonnet D Chapman SC Furbank RT Manes Y Mather DE Parry MAJ 2010 Raising yield potential of wheat. I. Overviews of a consortium approach and breeding strategies. Journal of Experimental Biology 62, 439–452 10.1093/jxb/erq311 20952629

[CIT0029] Reynolds MP Rajaram S Sayre KD 1999 Physiological and genetics changes of irrigated wheat in the post-green revolution period and approaches for meeting projected global demand. Crop Science 39, 1611–1621

[CIT0030] Reynolds MP van Ginkel M Ribaut J-M 2000 Avenues for genetic modification of radiation use efficiency in wheat. Journal of Experimental Biology 51, 459–473 10.1093/jexbot/51.suppl_1.459 10938854

[CIT0031] Richards RA 2000 Selectable traits to increase crop photosynthesis and yield of grain crops. Journal of Experimental Botany 51, 447–458 10938853 10.1093/jexbot/51.suppl_1.447

[CIT0032] Rincker K Nelson R Specht J 2014 Genetic improvement of US soybean in maturity groups II, II, and IV. Crop Science (E-pub ahead of print; 10.2135/cropsci2013.10.0665).

[CIT0033] Rowntree SC Suhre JJ Weidenbenner NH 2013 Genetic gain × management interactions in soybean: I. planting date. Crop Science 53, 1128–1138

[CIT0034] Sayre KD Rajaram S Fischer RA 1997 Yield potential progress in short bread wheat in northwest Mexico. Crop Science 37, 36–42

[CIT0035] Shearman VJ Sylvester-Bradley R Scott RK Foulkes MJ 2005 Physiological processes associated with wheat yield progress in the UK. Crop Science 45, 175–185

[CIT0036] Sinclair TR Muchow RC 1999 Radiation use efficiency. Advances in Agronomy 65, 215–265

[CIT0037] Slafer GA Andrade FH Storre EH 1990 Genetic improvement effects on pre-anthesis physiological attributes related to wheat grain yield. Field Crops Research 23, 225–263

[CIT0038] Slattery RA Ainsworth EA Ort DR 2013 A meta-analysis of responses of canopy photosynthetic conversion efficiency to environmental factors reveals major causes of yield gap. Journal of Experimental Botany 64, 3723–3733 23873996 10.1093/jxb/ert207 PMC3745731

[CIT0039] Specht JD Hume JD Kumudini SV 1999 Soybean yield potential—a genetic and physiological perspective. Crop Science 39, 1560–1570

[CIT0040] Tilman D Balzer C Hill J Befort BL 2011 Global food demand and the sustainable intensification of agriculture. Proceedings of the National Academy of Sciences, USA 108, 20260–20264 10.1073/pnas.1116437108 PMC3250154 22106295

[CIT0041] Zhu X-G Long SP Ort DR 2008 What is the maximum efficiency with which photosynthesis can convert solar energy into biomass. Current Opinion in Biotechnology 19, 153–159 18374559 10.1016/j.copbio.2008.02.004

[CIT0042] Zhu X-G Long SP Ort DR 2010 Improving photosynthetic efficiency for greater yield. Annual Review of Plant Biology 61, 235–261 10.1146/annurev-arplant-042809-112206 20192734

