# Supplementary material for: Historical gains in soybean (Glycine max Merr.) seed yield are driven by linear increases in light interception, energy conversion, and partitioning efficiencies
Source: J Exp Bot. 2014 Apr 30;65(12):3311–21. doi: 10.1093/jxb/eru187 (PMC4071847; doi:10.1093/jxb/eru187)
Supplement: Supplementary Data [file supp_65_12_3311__index.html]

Historical gains in soybean (Glycine max Merr.) seed yield are driven by linear increases in light interception, energy conversion, and partitioning efficiencies — Historical gains in soybean (Glycine max Merr.) seed yield are driven by linear increases in light interception, energy conversion, and partitioning efficiencies — Supplementary Data 

# Historical gains in soybean (*Glycine max* Merr.) seed yield are driven by linear increases in light interception, energy conversion, and partitioning efficiencies

## Supplementary Data

Data files

**Files in this Data Supplement:**

- Supplementary Data - Supplementary Data
