# Supplementary material for: Historical gains in soybean (Glycine max Merr.) seed yield are driven by linear increases in light interception, energy conversion, and partitioning efficiencies
Source: J Exp Bot. 2014 Apr 30;65(12):3311–21. doi: 10.1093/jxb/eru187 (PMC4071847; doi:10.1093/jxb/eru187)
Supplement: Supplementary Data [file supp_eru187_jexbot123141_file001.pdf]

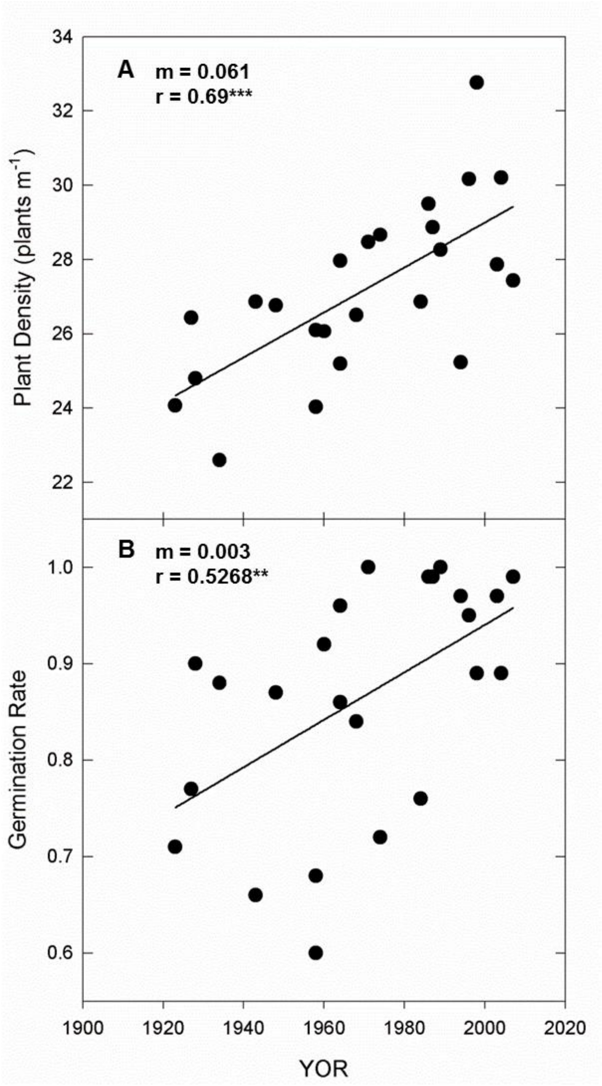

Figure S1. Plant density and seed germination versus YOR in 2011. Planting density (A) and germination rate (B) plotted against cultivar YOR. All lines represent the least squares regression (**p < 0.01, *** p < 0.001).

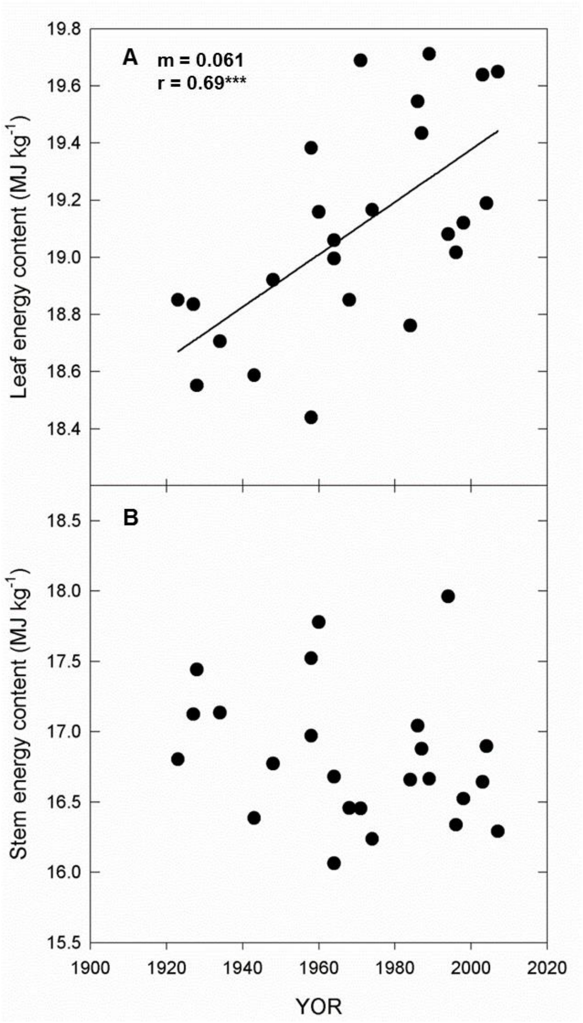

Figure S2. Leaf and stem energy content versus YOR. Leaf and stem energy content is plotted against cultivar YOR with the line representing the least squares regression (*** $p < 0.001$).

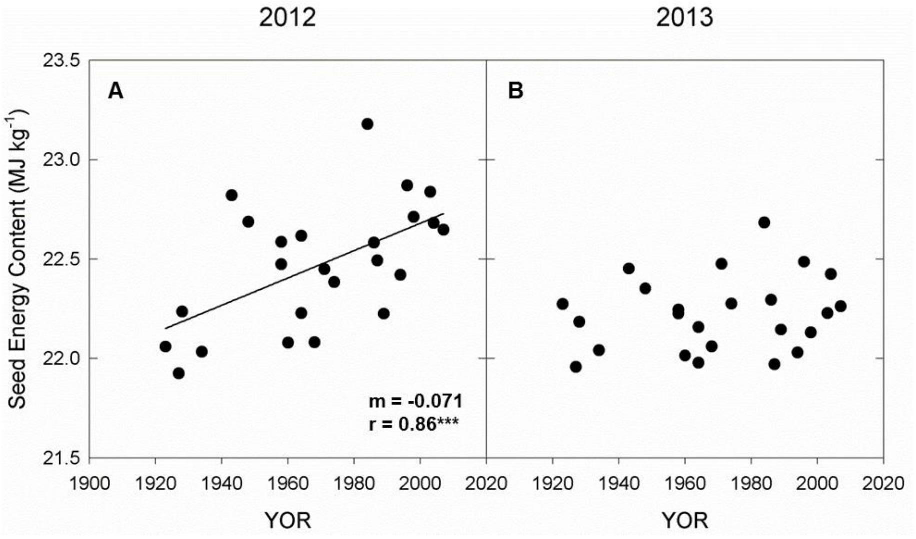

Figure S3. Seed composition versus YOR in 2012 and 2013. The energy content (A, B) of the seed is shown plotted against YOR in 2012 and 2013. All lines represent the least squares regression (*** $p < 0.001$).

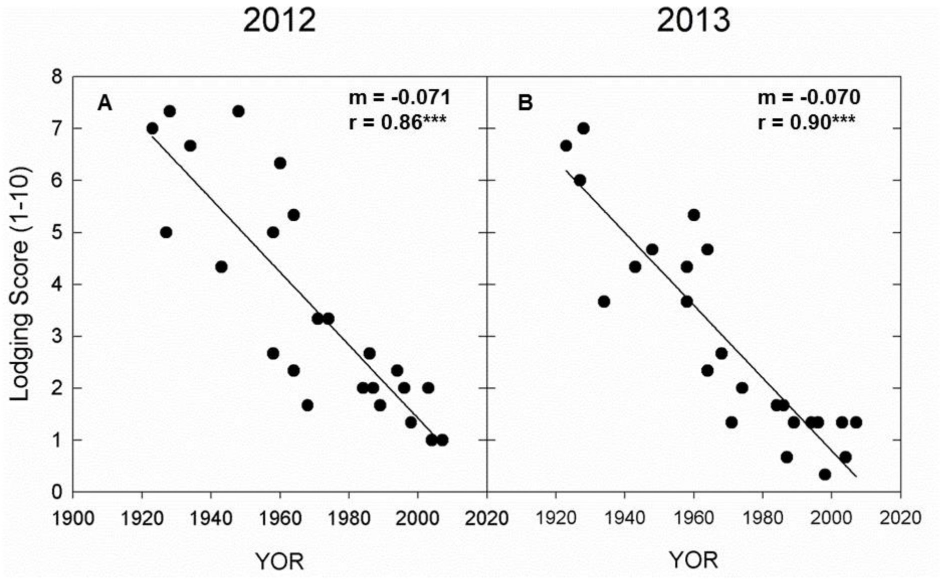

Figure S4. Lodging score versus YOR in 2012 and 2013. Lodging score is plotted against cultivar YOR with the line representing the least squares regression (*** $p < 0.001$). Each point is the average of three replicates.
